# CO_2_ Capture by Hyperbranched Poly(alkylene imine) Thin Films: A Molecular Dynamics Simulation Approach

**DOI:** 10.1021/acs.jpcb.6c02846

**Published:** 2026-08-26

**Authors:** Guilherme R. Weber Nakamura, Junhe Chen, Sung Hyun Kwon, Seung Soon Jang

**Affiliations:** † Computational NanoBio Technology Laboratory, School of Materials Science and Engineering, 1372 Georgia Institute of Technology, 771 Ferst Drive NW, Atlanta, Georgia 30332-0245, United States; ‡ School of Chemical Engineering, 34996 Pusan National University, 2 Busandaehak-ro 63beon-gil, Geumjeong-gu, Pusan 46241, South Korea

## Abstract

Direct air capture (DAC) of CO_2_ using solid amine sorbents requires a molecular-level understanding of interfacial phenomena under ultradilute conditions. In this study, we employ density functional theory (DFT)-calibrated force fields and molecular dynamics (MD) simulations to investigate CO_2_ capture behavior in thin films of hyperbranched poly­(ethylene imine) (HB-PEI) and poly­(propylene imine) (HB-PPI) using explicit slab geometries. The inclusion of a polymer–air interface induces significant structural changes, increasing the fractional free volume (FFV) from 19.1% to 25.0% for HB-PEI and from 19.9% to 29.9% for HB-PPI, along with increases in specific surface area to 0.29 and 0.34 Å^–1^, respectively. Despite these structural enhancements in HB-PPI, transport analysis reveals that HB-PEI consistently exhibits higher CO_2_ diffusivity, with diffusion coefficients decreasing from 0.937 × 10^–5^ to 0.049 × 10^–5^ cm^2^/s as CO_2_ and H_2_O loading increases, whereas HB-PPI shows lower values, ranging from 0.321 × 10^–5^ to 0.014 × 10^–5^ cm^2^/s. This indicates that CO_2_ transport is governed by polymer–gas interactions and segmental dynamics rather than free volume alone. Coordination number analysis further shows that HB-PEI exhibits strong CO_2_ affinity, with primary amine coordination decreasing from 0.655 to 0.464 under hydration, while HB-PPI displays an increase from 0.600 to 0.716, reflecting distinct interaction mechanisms. Additionally, pair correlation analysis reveals preferential CO_2_ binding to secondary amines in HB-PEI and primary amines in HB-PPI. Hydration introduces a critical limitation by forming hydrogen-bonded networks and competing for amine sites, leading to up to ∼95% reduction in CO_2_ diffusivity. These results demonstrate that interfacial heterogeneity, amine distribution, and hydration effects collectively govern CO_2_ capture behavior, underscoring the necessity of incorporating interfacial models for predictive design of DAC materials.

## Graphical abstract

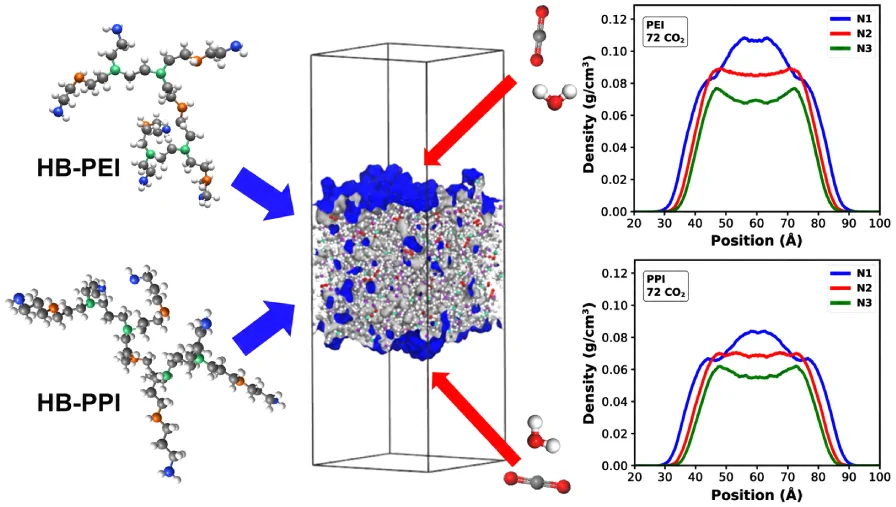

## 1 Introduction

Over the past century, the rapid increase in atmospheric CO_2_ concentration—primarily caused by human activity—has led to a rise in global temperatures exceeding 1 °C. To prevent global warming from surpassing 2 °C by the end of this century, both mitigating greenhouse gas emissions and actively removing CO_2_ from the air are essential, as emphasized by the Intergovernmental Panel on Climate Change (IPCC) and the Net Zero Emissions (NZE) roadmap.^1^ Achieving this goal requires large-scale deployment of carbon capture and storage (CCS) technologies, which remove CO_2_ either from industrial exhaust streams or directly from ambient air through direct air capture (DAC).^2^

However, it was noted that DAC poses a greater challenge due to the extremely low atmospheric CO_2_ concentration (∼0.04%);^2^ furthermore, within the framework of the traditional postcombustion carbon capture relying on aqueous amine solutions,^3,4^ it should be stressed that the process requires high regeneration energy due to the large heat capacity of water,^5^ making conventional carbon capture and storage (CCS) processes energy-intensive.

In this context, solid sorbent-based adsorption systems have emerged as energy-efficient alternatives.^6,7^ Thus, a wide range of solid adsorbents, such as activated carbons,^8,9^ metal oxides,^10,11^ and amine-based systems,^2,12–20^ have been studied for CO_2_ adsorption from gas mixtures. In particular, amine-functionalized polymers have received considerable attention^2,14–20^ because their high density of amine groups enables chemisorption through carbamate and bicarbonate formation, resulting in excellent CO_2_ selectivity.^21^ More importantly, compared to physisorbents such as metal–organic frameworks^22^ or zeolites,^8^ amine-based sorbents offer distinct advantages, including stronger binding energies, greater tolerance to humidity, and tunable regeneration properties.^2,23^ Commercial facilities such as Climeworks’ Orca and Mammoth plants in Iceland have demonstrated the feasibility of solid-amine DAC systems.^24,25^ While surface area is often associated with CO_2_ uptake, several studies have shown that the number of accessible amine sites is a more critical factor at ultralow CO_2_ concentrations (<1 vol %).^2,26^

To enhance the performance of solid amine DAC, various strategies, including physical impregnation,^13,27^ covalent tethering,^23,28^ in situ polymerization,^27,29^ and their combinations,^30^ have been utilized to incorporate amine functionalities into porous support materials. Among these, the impregnation method involves depositing amine species onto the surface or within the pores of the solid support, enabling the incorporation of amine moieties without forming covalent bonds to the material surface.^2^ Low-molecular-weight poly­(ethylenimine) (PEI) has been employed in numerous studies as an amine-containing polymer for CO_2_ adsorption due to its high density of amine groups and good performance under temperature swing adsorption (TSA) or vacuum swing adsorption (VSA) conditions.^6,7^ However, PEI is susceptible to oxidative degradation at high temperatures, which diminishes its long-term efficiency.^31^ In this context, another polymer, poly­(propylene imine) (PPI), has attracted attention.^32,33^

The chemical structure of PPI is similar to that of PEI, while its stability against oxidation is better than that of PEI,^32^ implying that PPI has a potentially longer operational lifetime than PEI. PPI possesses structural similarities to PEI, containing primary and secondary amines that interact with CO_2_ to form adsorbed species. Although both polymers undergo similar mechanisms^2^ of carbamate and bicarbonate formation upon exposure to CO_2_, variations in backbone rigidity, free volume, and amine accessibility impart distinct adsorption and diffusion characteristics. Because HB-PEI is already deployed at commercial scale in direct air capture facilities such as Climeworks’ Orca plant, yet remains limited by its susceptibility to oxidative degradation, while HB-PPI offers a closely related amine chemistry with markedly improved oxidative stability, we selected this pair specifically to isolate how backbone chemistry and oxidative stability govern CO_2_ capture performance, transport, and long-term sorbent durability at the molecular level.

In our recent work, we systematically investigated bulk hyperbranched PEI and PPI using molecular dynamics (MD) simulations with a DFT-calibrated force field (FF).^20^ We found that HB-PEI exhibits higher CO_2_ diffusivity and uptake than HB-PPI due to its greater amine density and more flexible backbone, which promote segmental motion and transient free-volume formation. However, we also found that hydration reduces CO_2_–amine coordination across both polymers by competing for adsorption sites and forming hydrogen-bonded water networks, thereby reducing the propensity for CO_2_ near amine sites to form carbamates. Although these insights clarified essential structure–property relationships in bulk systems, it is important to recognize that CO_2_ capture initiates at the surface of HB-PEI or HB-PPI. The investigation of the bulk phase did not account for surface-mediated mechanisms in practical DAC sorbents, highlighting the necessity of incorporating the surface into the analysis.

From this perspective, explicitly incorporating surface effects is essential to developing a physically realistic understanding of CO_2_ capture in hyperbranched poly­(alkylene imine)-based sorbents. In practical DAC operation, CO_2_ molecules first encounter the sorbent at the polymer–air interface, where surface composition, free volume, and molecular orientation govern initial adsorption, molecular partitioning, and subsequent diffusion into the bulk. In hyperbranched poly­(alkylene imine)­s such as HB-PEI and HB-PPI, the hyperbranched architectural topology drives nanoscale spatial heterogeneity across the polymer interface. Specifically, as demonstrated by the density profile analysis presented in Figure 3c and 3d, primary, secondary, and tertiary amine groups exhibit distinct density profiles along the direction normal to the interface, resulting in nanometer-scale spatial segregation, compositional gradients, and chain reorganization at the free surface. These surface-driven effects can strongly influence CO_2_ accessibility to reactive amine sites, competitive adsorption with H_2_O, and the overall transport kinetics that control capture efficiency under ultradilute conditions relevant to DAC.

However, it should be noted that despite extensive experimental and computational studies on bulk amine-loaded sorbents, direct insight into surface-mediated phenomena remains limited. Experimentally, techniques such as infrared spectroscopy, microcalorimetry, and neutron scattering have provided valuable information on amine–CO_2_ interactions and hydration effects, yet resolving molecular-scale distributions and transport pathways at polymer interfaces remains challenging. Similarly, numerous theoretical and simulation studies have focused on bulk polymer phases or polymer–support composites. Only a limited number of molecular simulations have addressed thin-film or slab geometries, even though such models are essential for capturing interfacial density gradients, surface enrichment of functional groups, and anisotropic diffusion near interfaces.^32,34,35^

In this study, we extend our previous molecular dynamics investigation^20^ of bulk HB-PEI and HB-PPI to explicitly examine the CO_2_ capture behavior in thin-film systems that include a polymer-gas phase interface. By employing a DFT-calibrated FF, we accurately describe the intermolecular interactions among CO_2_, H_2_O, and various amine functionalities present in the hyperbranched polyamines. Both three-dimensional bulk and two-dimensional slab models are constructed, enabling a direct comparison of structural, thermodynamic, and transport properties in the presence and absence of surfaces.

The primary objective of this study is to elucidate how the presence of a free surface in HB-PEI and HB-PPI thin films affects the molecular-level distribution and transport of CO_2_ compared to bulk systems. By revealing surface-specific effects that cannot be captured in bulk-only simulations, this work provides mechanistic insights into the early stages of CO_2_ capture. More broadly, our results highlight the necessity of incorporating interfacial models in molecular simulations of polymer-based gas-capture materials, particularly in regimes where transport and accessibility rather than equilibrium binding alone govern overall performance.

## 2 Models and Simulation Methods

### 2.1 Molecular Models and Force Fields for HB-PEI and HB-PPI

Atomistic models of HB-PEI (Figure 1a) and HB-PPI (Figure 1b) were constructed using experimentally reported primary:secondary:tertiary amine ratios of 6:5:4.^36^ The molecular weights of one hyperbranched unit were 619.98 g·mol^–1^ for HB-PEI and 816.35 g·mol^–1^ for HB-PPI. Each polymer structure was first geometry optimized using the density functional theory method (B3LYP/6-31G**) in Jaguar.^37^ Atomic partial charges were assigned using Mulliken population analysis for electrostatic interactions in MD simulations.

**Figure 1:**
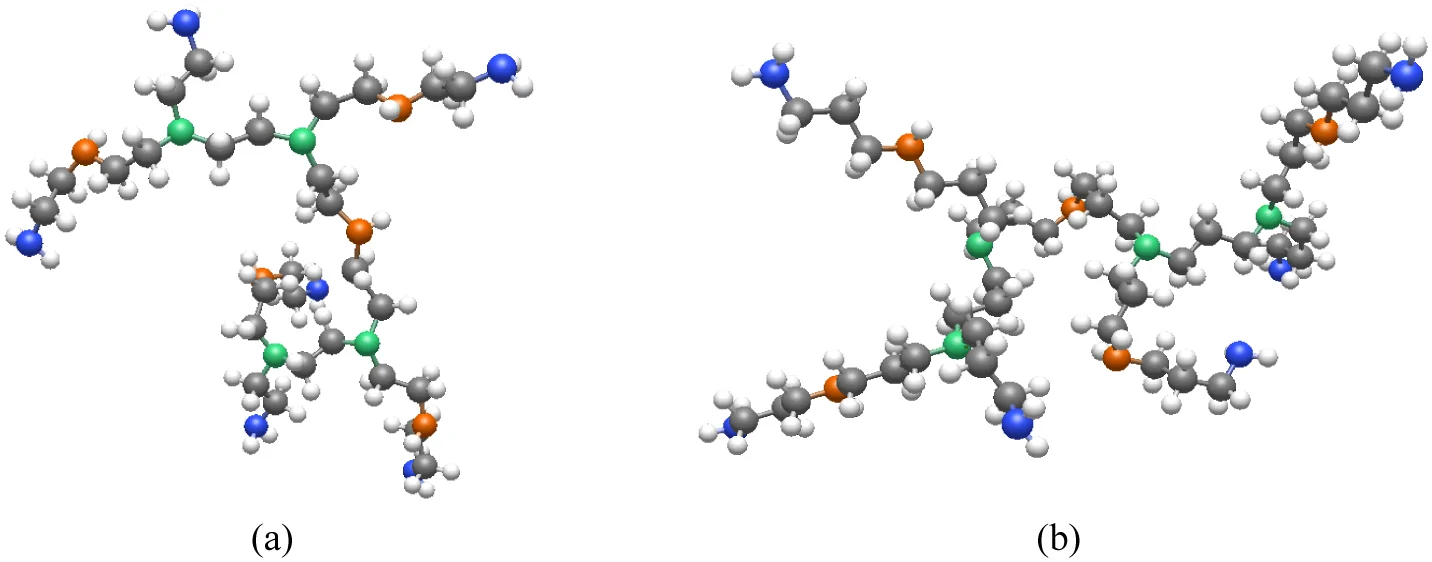
Molecular models for (a) hyperbranched poly­(ethylenimine) (HB-PEI) and (b) hyperbranched poly­(propylene imine) (HB-PPI). Blue, orange, and green denote nitrogen atoms in primary, secondary, and tertiary amines , respectively, and gray and white denote carbon and hydrogen, respectively.

Intramolecular and intermolecular interactions in our MD simulations were described using the DREIDING force field, while water molecules were modeled using the F3C water force field.^38,39^ The DREIDING force field has been well employed in numerous polymer simulation studies,^40–42^ and its results have been experimentally validated.^19,43–46^ The DREIDING force field has the following form:

1 $$E_{total}=E_{vdW}+E_{Q}+E_{bond}+E_{angle}+E_{torsion}+E_{inversion}$$

where *E_total_*, *E_vdW_*, *E_Q_*, *E_bond_*, *E_angle_*, *E_torsion_*, and *E_inversion_* are the total, van der Waals, electrostatic, bond-stretching, angle-bending, torsional, and inversion energies. *E_Q_* is calculated from atomic charges obtained from Mulliken population analysis.

In particular, to accurately describe interactions between amine–CO_2_, amine–H_2_O, and CO_2_–H_2_O pairs, we computed the energy as a function of distance using DFT with the B3LYP-D3 functional and the 6-31G** basis set, and then determined the Lennard–Jones (LJ) potential parameters (*D* and *r*_0_) for the off-diagonal van der Waals interaction $\left(E_{off-diagonal}\right)$ .^20^

### 2.2 Equilibrium MD Simulations of HB-PEI and HB-PPI

To simulate the slab systems of HB-PEI and HB-PPI, first, we prepared equilibrated 3-dimensional amorphous systems with periodic boundary conditions in all directions, as reported in our previous study,^20^ and then extended the cell dimension in *z*-axis direction to create empty space above and below the slab of HB-PEI and HB-PPI, as shown in Figure 2, so that the simulation box contains a slab in the *x*- and *y*-axis directions under periodic boundary conditions, except for *z*-axis direction. The numbers of molecules in the simulated systems are summarized in Table 1.

**Figure 2:**
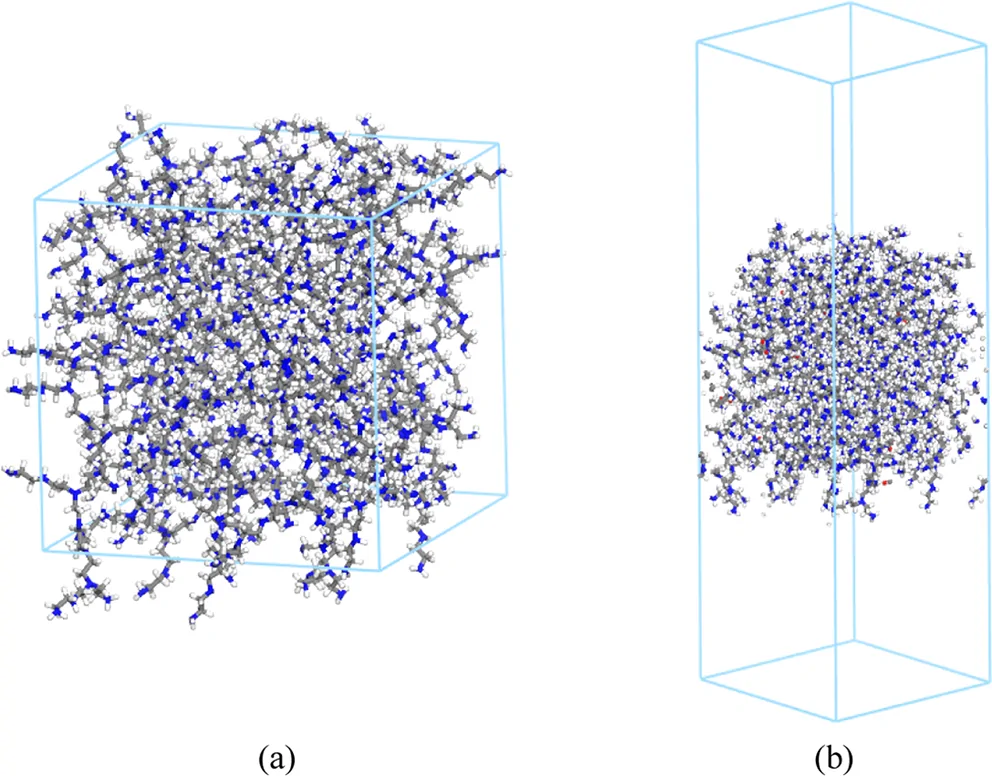
Simulated structures of HB-PEI: (a) 3D amorphous bulk phase; (b) 2D amorphous slab structure. HB-PPI structures have similar features.

**Table 1:** Details of the Simulated Systems

Polymer Types (Number of Polymers)	System Codes	Number of CO_2_	Number of H_2_O	Density (g/cm^3^)
HB-PEI (50)	PEI-0-0	0	0	0.504
	PEI-12-0	12	0	0.507
	PEI-36-0	36	0	0.522
	PEI-72-0	72	0	0.531
	PEI-72-36	72	36	0.544
	PEI-72-72	72	72	0.567
	PEI-72-144	72	144	0.597
HB-PPI (38)	PPI-0-0	0	0	0.488
	PPI-12-0	12	0	0.492
	PPI-36-0	36	0	0.510
	PPI-72-0	72	0	0.521
	PPI-72-36	72	36	0.539
	PPI-72-72	72	72	0.560
	PPI-72-144	72	144	0.582

It is important to clarify that each hyperbranched unit modeled here comprises approximately 14 monomeric repeat units, corresponding to an oligomer rather than an infinitely long polymer chain. The polymer phase in our simulation is constructed by packing multiple such hyperbranched oligomer units, as in experiments, and serves as a molecular representation designed to capture the local packing characteristics and transport behavior of the experimentally observed hyperbranched architecture at a similar molecular weight.^32,47^ It should be noted that the use of oligomers has some limitations: it does not permit long-chain entanglement or long-range motion. This can lead to drawbacks with respect to mechanical properties. Nevertheless, short-range packing, intermolecular interactions, transport, and permeability can be reliably simulated, as they have been extensively studied in the literature.^19,20,36^

After preparing the initial slab structures, we performed MD simulations using the LAMMPS software.^45^ To achieve equilibrium in the simulated systems, we used the thermal annealing procedure outlined by Jang and Goddard,^48^ which accelerates equilibration by providing additional kinetic energy through repetitive thermal cycling to overcome energy barriers associated with trapped structures.^48^ Please note that no particular molecular or packing structures for HB-PEI and HB-PPI were assumed during the annealing procedure. Then, we performed an NVT MD simulation for 60 ns at T = 303.15 K. After completing the equilibrium MD simulation, we used the last 10 ns segment of the trajectory file for data analysis.

### 2.3 Density Profile

To quantitatively characterize the distribution of molecules at the polymer–air interface in the slab systems, we obtained the density profile, ρ­(*z*), by binning all atoms along the *z*-axis. The resulting profile typically shows a plateau in the bulk region and a gradual decay to zero near the interface. We then employed eq 2:

2 $$\rho \left(z\right)=\frac{\rho_{\mathrm{bulk}}}{2}\left[1-\tanh\left(\frac{z-z_{0}}{d}\right)\right]$$

where *ρ*_bulk_is the bulk density in the interior of the polymer slab, *z*_0_ is the interface position, defined as the location where the density falls to half of *ρ*_bulk_, and *d* is a measure of the interfacial thickness, reflecting how rapidly the density transitions from bulk to air.

This method provides a robust and smooth way to identify the interfacial boundary in systems with diffuse interfaces, which are not well captured by simple thresholding. Nonlinear least-squares fitting is performed on the density data using the above equation, treating *ρ*_bulk_, *z*_0_, and *d* as fitting parameters. The quality of the fit is typically excellent, since the hyperbolic tangent function accurately captures the sigmoidal decay of the density profile near surfaces.

### 2.4 Free Volume and Specific Surface Area

In contrast to the bulk-phase analysis reported in our previous study,^20^ in which the free volume was inferred primarily from bulk density evolution under periodic boundary conditions, the slab systems in this study require explicit spatial identification of void regions along the nonperiodic *z*-axis. Thus, the free volume (*V_Free_*) was calculated using the probe-accessible volume approach. Equilibrated polymer configurations were discretized onto a three-dimensional grid, and a spherical probe with a radius equivalent to that of a CO_2_ molecule was systematically inserted throughout the simulation box, with grid points that did not overlap with any polymer atoms, based on van der Waals radii, classified as accessible void space. Then, the fractional free volume (*FFV*) is defined as *FFV* = *V_Free_*/*V_Slab_*, where *V_Slab_* denotes the slab volume within the interfacial boundaries of the density profiles. This definition ensures that the calculated free volume reflects the intrinsic polymer porosity.

## 3 Results and Discussion

### 3.1 Structural Analysis of HB-PEI and HB-PPI in Bulk and Slab Systems

#### 3.1.1 Density Profile

By equilibrating the initial structures of the slab systems for HB-PEI and HB-PPI through NVT MD simulations, we characterized the density profiles by slicing the slab into 1-Å-thick slices and counting the atoms in each slice. Figure 3a and 3b presents the typical density profiles for the HB-PEI and HB-PPI systems, respectively, while Figure 3c and 3d presents the component density profiles of primary, secondary, and tertiary amines for the HB-PEI and HB-PPI systems, respectively, and Figure 3e and 3f presents the component density profiles of CO_2_ and H_2_O for the HB-PEI and HB-PPI systems, respectively.

**Figure 3:**
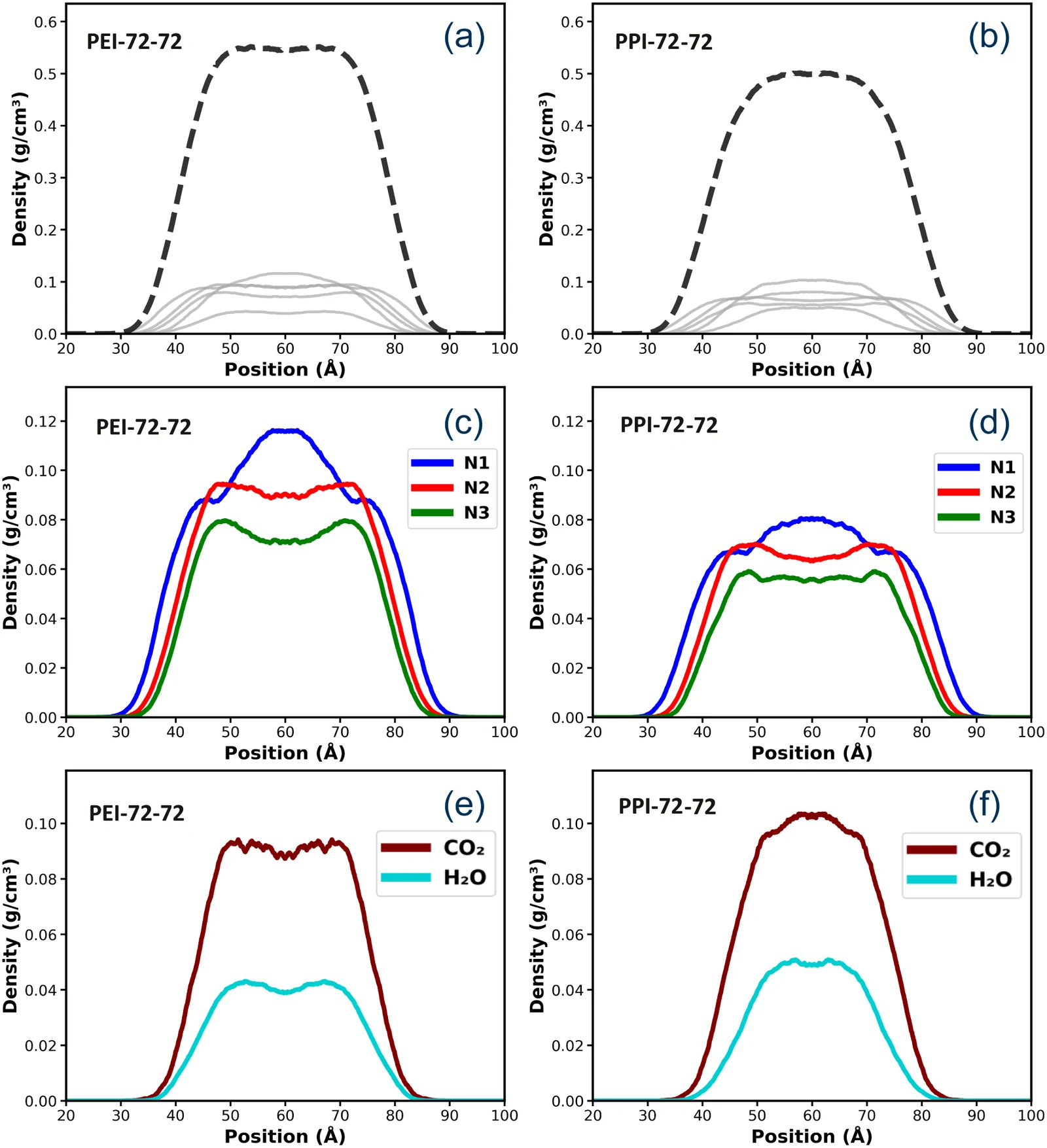
Density profiles of PEI-72-72 and PPI-72-72 systems: (a) and (b) total density profiles; (c) and (d) distributions of primary, secondary, and tertiary amines; (e) and (f) distributions of CO_2_ and H_2_O.

Figure 3a and 3b shows that HB-PEI has a higher overall density than HB-PPI, which is attributed to the higher amine density of HB-PEI, as shown in Figure 3c and 3d. These density profiles indicate that HB-PPI has less packing than HB-PEI does. As CO_2_ and H_2_O are added, the density increases, as expected.

Interestingly, Figure 3c and 3d shows that the primary, secondary, and tertiary amines present very distinct distributions in the slab system. The primary amines tend to aggregate toward the middle of the system, whereas the secondary amines are flatter with no clear preference, and the tertiary amines tend toward the surface. This phenomenon can be explained by the greater hydrophilicity of primary amines, which prefer to remain within the polymer, closer to H_2_O and CO_2_ groups in the bulk and farther from the air or vacuum. Following this trend, as hydrophilicity decreases, the secondary amines exhibit intermediate behavior and a balanced profile, while the tertiary amines are concentrated on the surface.

On the other hand, Figure 3e and 3f shows that CO_2_ is mostly present deep within the polymer. In particular, we note that CO_2_ has a higher concentration in HB-PPI than in HB-PEI. That aligns with our observation of less packing in HB-PPI than in HB-PEI, indicating that HB-PPI has a greater internal capacity to accommodate CO_2_ molecules. This is also evident from CO_2_ interacting more frequently with primary amines in HB-PPI than in HB-PEI, by comparing the amine profile with the CO_2_ profile. H_2_O exhibits similar behavior to CO_2_, aggregating toward the center.

#### 3.1.2 Free Volume and Specific Surface Area

Next, we characterized the free volume in the HB-PEI and HB-PPI slab systems, as shown in Figure 4, to understand how CO_2_ capture characteristics correlate with free volume; free volume can be interpreted as the available volume in the systems. The fractional free volume (*FFV)* and specific surface area (SSA) of HB-PEI and HB-PPI in both bulk and slab configurations are summarized in Table 2. These structural descriptors provide quantitative insights into polymer packing characteristics and interfacial accessibility, which are directly relevant to gas diffusion and adsorption processes.

**Figure 4:**
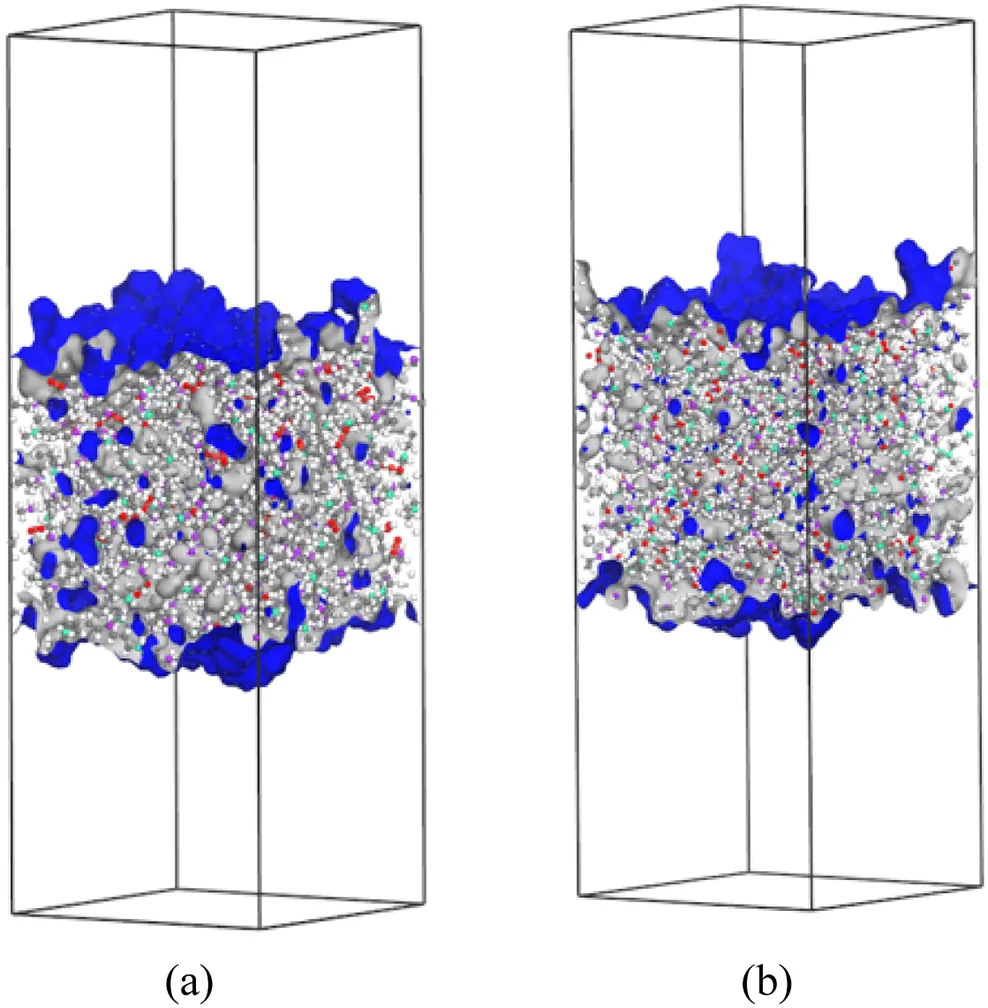
Free volume and specific surface in slab systems: (a) HB-PEI; (b) HB-PPI.

**Table 2:** Fractional Free Volume (FFV) and Specific Surface Area (SSA)

Phase	Polymer	Free Volume	Specific Surface Area (Å^–1^)
**Bulk**	HB-PEI	19.1%	0.27
	HB-PPI	19.9%	0.29
**Slab**	HB-PEI	25.0%	0.29
	HB-PPI	29.9%	0.34

In our study,^20^ the bulk phases of HB-PEI and HB-PPI exhibit comparable free volume fractions of 19.1% and 19.9%, respectively. The slightly higher FFV observed for HB-PPI is attributable to the presence of an additional methylene group in its backbone relative to HB-PEI, which reduces packing efficiency and increases segmental flexibility. This structural distinction is also reflected in the bulk SSA values, with HB-PPI (0.290 Å^–1^) exhibiting a modestly higher accessible surface area than HB-PEI (0.270 Å^–1^).

Upon transitioning from bulk to slab geometries, both HB-PEI and HB-PPI systems display a substantial increase in free volume. The FFV increases to 25.0% for HB-PEI and 29.9% for HB-PPI. This enhancement of FFV in both systems arises from the introduction of polymer–air interfaces, which reduce confinement in the surface-normal direction and allow further relaxation of chain segments near the interfaces. The magnitude of the increase is more pronounced for HB-PPI, consistent with its greater backbone flexibility and lower density values shown in the density profile (Figure 3). This result suggests that the presence of a free surface leads to more substantial structural reconfiguration in HB-PPI than in HB-PEI.

A corresponding increase in specific surface area is observed. In the slab configurations, the SSA increases to 0.293 Å^–1^ for HB-PEI and 0.338 Å^–1^ for HB-PPI. The elevated SSA reflects the formation of accessible interfacial regions that are absent in fully periodic bulk systems. The larger SSA of the HB-PPI slab indicates a greater extent of exposed polymer surface, which may influence sorbate accommodation and initial adsorption events.

Collectively, the free volume and surface area analyses demonstrate that the presence of a surface leads to measurable changes in the polymer morphology. The slab configuration amplifies the void space and surface accessibility, particularly in HB-PPI. These structural differences provide a mechanistic basis for interpreting variations in sorbate distribution and transport behavior, as discussed in the following sections.

### 3.2 Distribution of CO_2_ and H_2_O Molecules

#### 3.2.1 Pair Correlation Analysis

We employed the pair correlation function, *g*_*A*–*B*_(*r*), to quantify the distributions of CO_2_ and H_2_O relative to amine–CO_2_ and amine–H_2_O. *g*_*A*–*B*_(*r*) represents the probability density of finding atoms *A* and *B* at a distance *r*, averaged over the equilibrium trajectory, as shown in eq 3):

3 $$g_{A-B}\left(r\right)=\frac{n_{B}}{4\pi r^{2}dr}/\frac{N_{B}}{V}$$

where *n*_*B*_ is the number of *B* particles located at a distance *r* from particle *A* within a shell of thickness *dr*, *N*_*B*_ is the total number of *B* particles in the system, and *V* is the volume of the system. Note, however, that we used *ρ_B_* · *g_A–B_* (*r*) where *ρ_B_* is the number density of *B* particles (*ρ_B_* = *N_B_*/*V*) to quantitatively compare the pair correlation:

4 $$\rho_{B}\cdot g_{A-B}\left(r\right)=\frac{n_{B}}{4\pi r^{2}dr}$$

Consequently, *ρ_B_* · *g_A–B_* (*r*) represents the number density of *B* particles at a distance *r* from the *A* particle, allowing high and low number densities to be directly interpreted as strong and weak correlations, respectively.

As observed in the HB-PEI and HB-PPI bulk systems,^20^ Figures 5 and 6 show significant similarity in the pair correlations of HB-PEI and HB-PPI in the slab systems, attributable to their analogous structures, differing by merely one additional carbon in the backbone between the amines.

**Figure 5:**
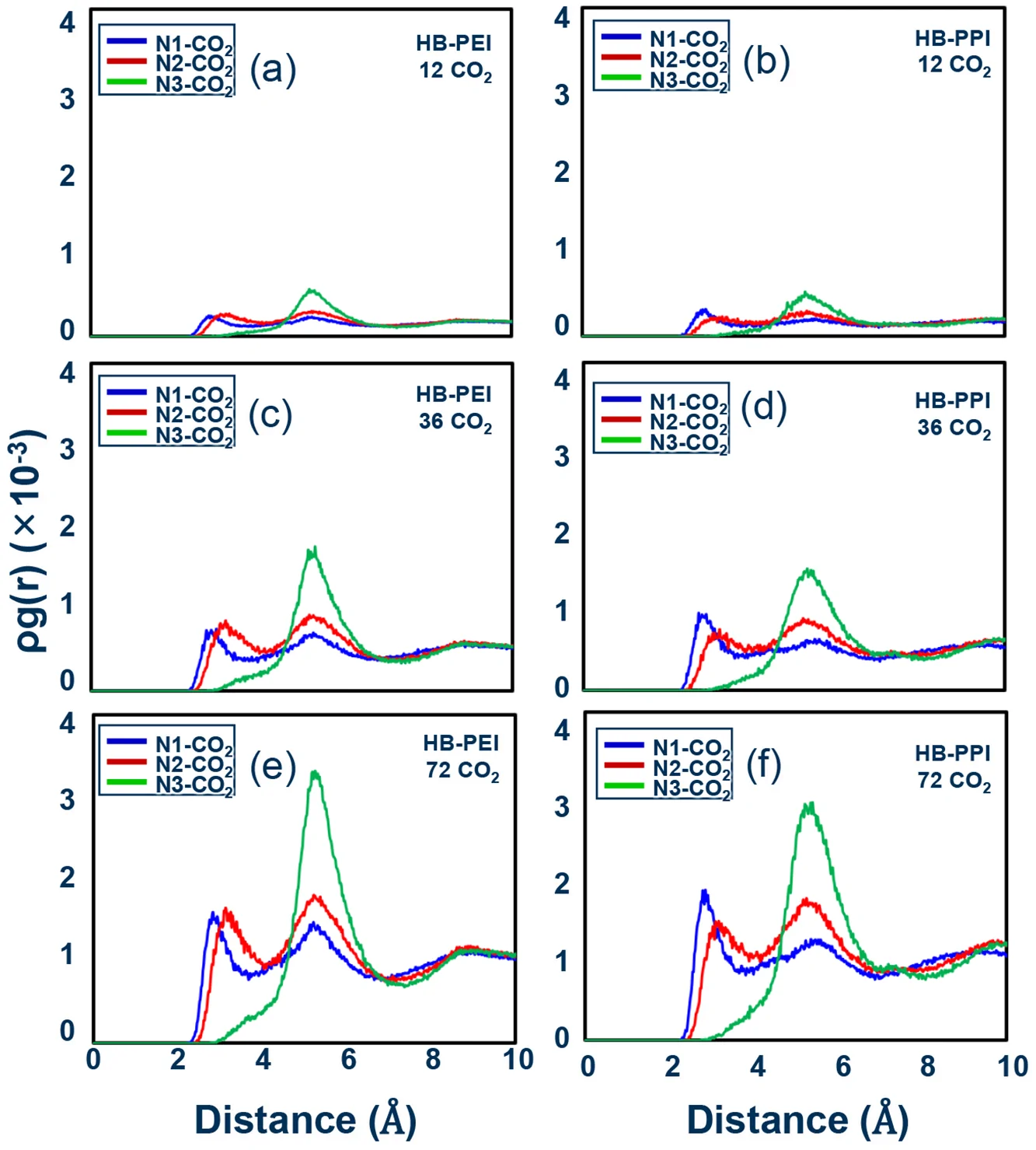
Pair correlation functions for amine–CO_2_ pairs in HB-PEI and HB-PPI slab systems in the absence of H_2_O molecules: (a, b) for PEI-12-0 and PPI-12-0, respectively; (c, d) for PEI-36-0 and PPI-36-0, respectively; and (e, f) for PEI-72-0 and PPI-72-0, respectively.

**Figure 6:**
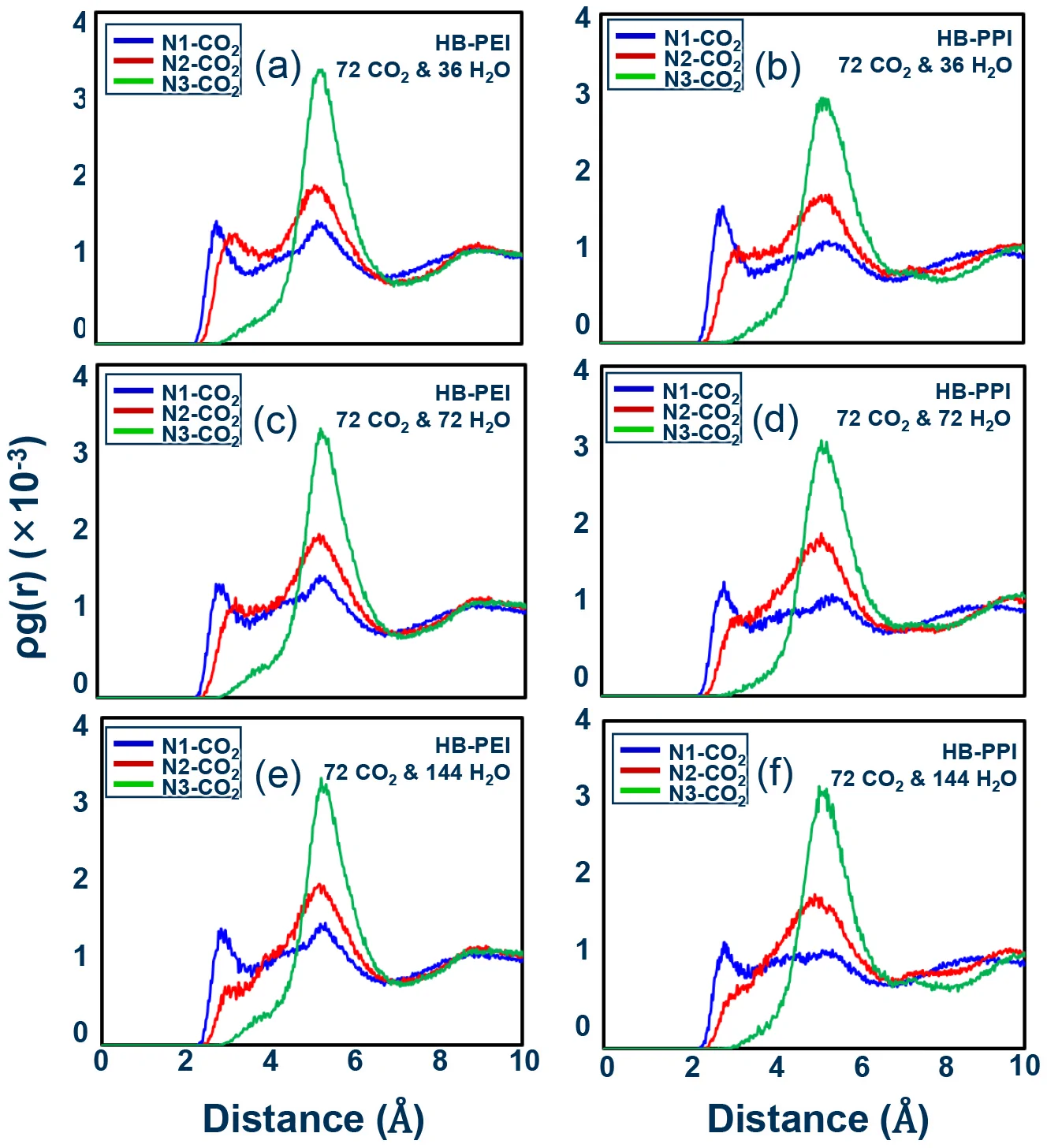
Pair correlation functions for amine–CO_2_ pairs in HB-PEI and HB-PPI slab systems in the presence of H_2_O molecules: (a, b) for PEI-72-36 and PPI-72-36, respectively; (c, d) for PEI-72-72 and PPI-72-72, respectively; and (e, f) for PEI-72-144 and PPI-72-144, respectively.

From Figure 5 for systems in the absence of H_2_O, it is observed that CO_2_ exhibits a stronger correlation with secondary amines (N2) in HB-PEI compared to HB-PPI, whereas CO_2_ demonstrates a greater correlation with primary amines (N1) in HB-PPI than in HB-PEI, indicating a distinct preference of CO_2_ for secondary amines in HB-PEI and primary amines in HB-PPI. Clearly, the augmentation of CO_2_ levels in both systems results in consistent increases in *ρg*(*r*).

On the other hand, Figure 6 shows that, with the addition of H_2_O molecules into the slab systems, HB-PEI and HB-PPI converge to similar behavior with considerably higher peaks for the primary amine–CO_2_ pair. Both systems exhibit a gradual reduction in the first peak for both primary and secondary amines as the number of H_2_O molecules increases; however, this effect is significantly more pronounced for secondary amines, suggesting that water displaces CO_2_, resulting in carbon interacting with the nitrogen atoms from a greater distance. Figure S1 shows that the *ρg*(*r*) for the amine–H_2_O pair also increases proportionally with H_2_O amount, which matches the decrease in the CO_2_ correlation peaks. In particular, the growth of the strongest peak for the secondary amine–H_2_O pair explains why the secondary amine–CO_2_ correlation is reduced.

#### 3.2.2 Coordination Number

Next, for quantitative analysis, we calculated the CO_2_ coordination number (CN) for each amine group (primary, secondary, and tertiary; Table 3) by integrating the first peak of the amine–CO_2_ pair correlation using eq 5):

5 $$CN=4\pi \int_{0}^{r_{first}}\rho g\left(r\right)r^{2}dr$$

**Table 3:** CO_2_ Coordination Numbers for Amine Groups in HB-PEI and HB-PPI Slab Systems

Systems	Number of CO_2_	Number of H_2_O	$CN_{CO_{2}}$ (1N)	$CN_{CO_{2}}$ (2N)	$CN_{CO_{2}}$ (3N)
**HB-PEI**	12	0	0.08	0.06	0.07
	36	0	0.32	0.07	0.41
	72	0	0.66	0.28	0.69
	72	36	0.52	0.20	0.68
	72	72	0.57	0.29	0.67
	72	144	0.46	0.60	0.66
**HB-PPI**	12	0	0.05	0.08	0
	36	0	0.26	0.11	0.29
	72	0	0.60	0.13	0.64
	72	36	0.62	0.30	0.30
	72	72	0.64	0.74	0.60
	72	144	0.72	0.68	0.49

In this context, the coordination number is the number of CO_2_ molecules within the solvation shell of amine groups. This number does not directly represent the true amine efficiency because classical molecular dynamics studies cannot account for chemical reactions. Thus, we cannot directly determine the stoichiometric adsorption efficiency from this. Instead, it represents molecular association, which correlates with the potential for CO_2_ absorption. Table 3 shows that HB-PEI exhibits higher overall CN than HB-PPI under both dry and hydrated conditions, indicating a higher capture capacity for HB-PEI, despite HB-PPI having greater free volume.

For HB-PEI and HB-PPI without water, as shown in Table 3, the CN for primary, secondary, and tertiary amines starts at small values and steadily increases as more CO_2_ is added. This trend continues as the amount of CO_2_ in the system increases.

On the other hand, in hydrated conditions, HB-PEI and HB-PPI behave differently. In HB-PEI, there is a consistent decrease in CN for primary amines, from 0.655 to 0.464, and a nonmonotonic behavior for secondary amines: the CN first decreases from 0.284 to 0.198, then recovers to 0.288 and rises further to 0.602 at the highest hydration level. A 4.0 Å cutoff was applied to the CN for secondary amines, as the first and second solvation shells merge with the addition of water, which makes a tighter cutoff, based on other amines, necessary to isolate reasonable values for the first-shell coordination. The CN for tertiary amines, however, stays relatively constant across all hydration levels, ranging narrowly between 0.661 and 0.685. These trends reflect the intense competition between CO_2_ and H_2_O for primary and secondary amine sites, with water progressively displacing CO_2_ from the first coordination shell. The tertiary amine behavior supports the hypothesis that when CO_2_ is displaced from the main reactive sites, it remains associated with the sterically accessible tertiary amine sites.

In contrast, HB-PPI shows an increase in CN for the primary amine from 0.600 to 0.716 with increasing hydration. Examination of the pair correlation functions reveals a visible reduction in peak intensity; nevertheless, the profile becomes more uniform overall, which leads to a net increase in the integrated CN. For secondary amines in HB-PPI, the 4.0 Å cutoff was likewise required due to solvation shell fusion, and the resulting CN values show a nonmonotonic but generally increasing trend from 0.130 to 0.679. The more moderate displacement compared with HB-PEI suggests that amine–CO_2_ interactions in HB-PPI are less sensitive to hydration, consistent with the more hydrophobic character and lower amine density of HB-PPI. The tertiary amines decrease from 0.636 to 0.489 owing to changes in the shape of the step preceding the large solvation shell, while the shell itself remains structurally stable.

These patterns are consistent with the established understanding that primary and secondary amines attract CO_2_ through hydrogen bonding or chemisorb CO_2_ through nucleophilic attack, thereby prioritizing interactions with CO_2_, whereas tertiary amines follow the bicarbonate mechanism, which inherently involves H_2_O.

### 3.3 Diffusivity of CO_2_ and H_2_O

Efficient CO_2_ capture in the HB-PEI and HB-PPI slab systems relies heavily on the transport of CO_2_ and H_2_O molecules, as poor transport can impede adsorption–desorption and negatively affect overall performance. To quantify the transport behavior, we analyzed the mean-square displacement (MSD) of CO_2_ and H_2_O molecules over time as defined in eq 6:

6 $$MSD=\frac{1}{N}\overset{N}{\underset{i=1}{\sum }}{|r\left(t\right)-r\left(0\right)|}^{2}$$

where ***r*** (*t*) and ***r*** (0) denote the positions of particle *i* at time *t* and at the beginning, *N* denotes the number of particles. Figure 7 shows only the PEI-72-0, PEI-72-36, PPI-72-0, and PPI-72-36 systems as examples, since all simulated systems exhibit similar MSD behavior. Accordingly, the overall diffusion coefficient (*D*) was evaluated using eq 7:

7 $$D=\frac{1}{6N}\underset{t\to \infty }{\lim}\frac{1}{t}\overset{N}{\underset{i=1}{\sum }}{|r_{i}\left(t\right)-r_{i}\left(0\right)|}^{2}=\frac{1}{6}\underset{t\to \infty }{\lim}\frac{1}{t}MSD$$

**Figure 7:**
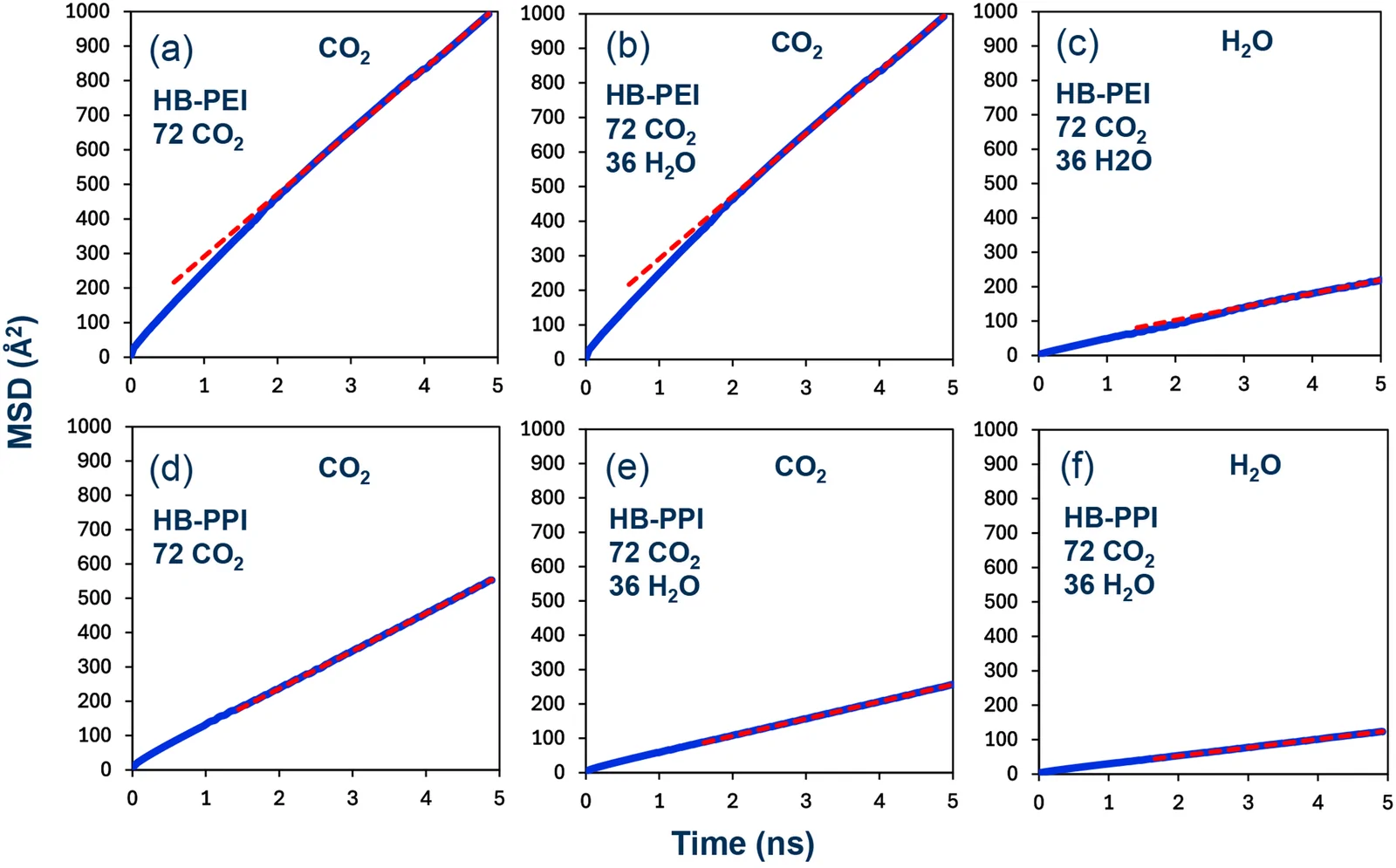
Mean-square displacement for CO_2_ and H_2_O in HB-PEI and HB-PPI slab systems: (a, d) for CO_2_ in the absence of H_2_O; (b, e) for CO_2_ in the hydrated systems; (c, f) for H_2_O in the presence of CO_2_ in the hydrated systems.

Using this approach, we calculated diffusion coefficients, as summarized in Table 4 for CO_2_ and H_2_O and, furthermore, for primary, secondary, and tertiary amines.

**Table 4:** Diffusion Coefficients of CO_2_, H_2_O, and Amine Groups in Slab Systems

			Diffusion Coefficient (D_s_)		
Polymer	Number of CO_2_	Number of H_2_O	CO_2_ (×10^–5^ cm^2^/s)	H_2_O (×10^–5^ cm^2^/s)	Amine (N) (×10^–5^ cm^2^/s)
**HB-PEI**	12	0	0.937 ± 0.005	/	0.104
	36	0	0.524 ± 0.002	/	0.106
	72	0	0.311 ± 0.002	/	0.121
	72	36	0.316 ± 0.001	0.072 ± 0.002	0.108
	72	72	0.125 ± 0.001	0.068 ± 0.002	0.091
	72	144	0.049 ± 0.001	0.019 ± 0.001	0.104
**HB-PPI**	12	0	0.321 ± 0.010	/	0.086
	36	0	0.262 ± 0.022	/	0.075
	72	0	0.181 ± 0.009	/	0.099
	72	36	0.082 ± 0.001	0.040 ± 0.001	0.102
	72	72	0.074 ± 0.001	0.038 ± 0.001	0.093
	72	144	0.014 ± 0.003	0.028 ± 0.001	0.086

The tabulated diffusion coefficients reveal systematic trends in the transport properties of CO_2_, H_2_O, and the amines. For HB-PEI, the CO_2_ diffusion coefficient decreases regularly from 0.937 × 10^–5^ cm^2^/s at 12 CO_2_ molecules to 0.311 × 10^–5^ cm^2^/s at 72 CO_2_ molecules, in the absence of water. Then, in the hydrated systems, it is further reduced to 0.049 × 10^–5^ cm^2^/s, indicating a significant reduction in transport with increasing CO_2_ and H_2_O loading. The introduction of water results in a more significant decrease in CO_2_ diffusivity, suggesting that H_2_O molecules occupy free volume or form hydrogen-bonding networks that impede CO_2_ transport. HB-PPI exhibits qualitatively similar behavior but with consistently lower baseline CO_2_ diffusion coefficients (0.321 × 10^–5^ cm^2^/s at 12 CO_2_/0 H_2_O, decreasing to 0.014 × 10^–5^ cm^2^/s at 72 CO_2_/144 H_2_O), indicating a less permeable polymer matrix compared to HB-PEI. This result seems unreasonable at first glance because HB-PPI has a higher free volume, which could indicate greater ease of movement and, thus, a higher diffusion coefficient. Nevertheless, in a highly interacting environment with significant steric hindrance, thermal fluctuations prevail, as previously shown in bulk systems.^23^

This apparent inconsistency can be more rigorously understood by considering that diffusivity in these systems is governed by the coupling between transient free-volume connectivity and polymer–gas interactions rather than static free volume alone. In our previous bulk-phase study, we demonstrated that HB-PEI exhibits enhanced segmental mobility and dynamic free-volume fluctuations, which facilitate the formation of temporally connected diffusion pathways despite its slightly lower average free volume. In contrast, although HB-PPI possesses a higher fractional free volume (29.9% vs 25.0% in slab systems), its relatively more hydrophobic backbone and reduced amine density lead to weaker coupling between CO_2_ and polymer segmental motion, resulting in less efficient hopping between voids. Moreover, stronger and more frequent CO_2_–amine interactions in HB-PEI, particularly with secondary amines, enable a “binding–release–hopping” mechanism that enhances effective transport, whereas in HB-PPI, CO_2_ molecules tend to reside in larger but more isolated free-volume pockets, leading to increased residence time and reduced mobility. This effect becomes more pronounced under hydrated conditions, where hydrogen-bonded water networks further disrupt free-volume connectivity and introduce dynamic trapping, disproportionately affecting HB-PPI due to its less flexible network response. Therefore, these results highlight that dynamic heterogeneity, interaction-driven transport, and connectivity of accessible pathways collectively dominate CO_2_ diffusion, consistent with trends observed in our prior bulk simulations.

This trend is also consistent with experimental comparisons of the PEI- and PPI-based sorbents. Pang et al. compared silica-supported linear and dendritic PEI and PPI sorbents and found that regardless of amine loading or architecture, PPI-based materials achieved higher overall CO_2_ capture efficiency per amine site than PEI-based materials, in addition to improved oxidative stability.^47^ We note that this experimental capture efficiency reflects the equilibrium extent of carbamate/bicarbonate formation, which is governed by amine chemical reactivity and accessibility, whereas the diffusion coefficients reported here characterize the kinetics of molecular transport through the polymer matrix; the two are therefore complementary rather than contradictory measures of sorbent performance.

The diffusion coefficients of the amine groups are consistently lower than those of CO_2_, which is consistent with limitations arising from their covalent connection to the backbone and their strong interactions with the surrounding CO_2_ and H_2_O. Notably, the amine diffusivity decreases with increasing CO_2_ and H_2_O contents in both polymers, which is attributed to interactions of the amines with CO_2_ and H_2_O that further limit their mobility.

The water diffusivity shows a slight decrease with increasing concentration, possibly due to an increase in hydrogen-bonding interactions between water molecules. The presence of water molecules consistently reduces the mobility of all components, likely through hydrogen bonding and competition for free volume occupation. These trends are consistent with our previous study^2^ on bulk-phase systems; however, the slab systems exhibit an overall higher diffusivity and a less drastic reduction in diffusivity due to the presence of H_2_O.

This diffusion-limiting effect of water is corroborated by experimental kinetic studies of supported amine sorbents. Liu et al. developed a kinetic model for direct air capture and reported that the CO_2_ uptake rate constant decreased by roughly half under humid, compared to dry, conditions, attributing the slowdown to competitive occupation of amine sites and hindered CO_2_ diffusion through the hydrated amine layer.^49^ This is consistent with our observation that water molecules preferentially associate with amine groups and form competing hydrogen-bond networks that reduce CO_2_ mobility, even though the equilibrium CO_2_ uptake capacity of amine sorbents is often reported to increase with humidity through additional thermodynamic pathways not directly probed by our diffusion analysis.

### 3.4 Comparison of Slab against Bulk Systems

To better understand the unique interfacial behavior of the thin-film models, Table 5 presents the diffusion coefficients and coordination numbers of the slab systems in comparison with the bulk systems^20^ previously studied. This comparison reveals two major physical insights that are specifically captured by the slab geometry. First, the diffusion coefficients for CO_2_ are consistently higher in the slab models than in their bulk counterparts, regardless of polymer type (HB-PEI or HB-PPI) or hydration state. For instance, the diffusion coefficient of CO_2_ in dry HB-PEI increases from 0.179 ± 0.003 × 10^–5^ cm^2^/s in the bulk to 0.311 ± 0.002 × 10^–5^ cm^2^/s in the slab, confirming enhanced gas mobility at the unconstrained polymer interface. The same trend holds for HB-PPI, where the dry-state CO_2_ diffusion coefficient rises from 0.097 ± 0.002 × 10^–5^ cm^2^/s in the bulk to 0.181 ± 0.009 × 10^–5^ cm^2^/s in the slab.

**Table 5:** Comparison of Diffusion Coefficients and Coordination Numbers in Selected Bulk and Slab Systems

			Diffusion Coefficient (D_s_)		Coordination Number		
Polymer	Number of CO_2_	Number of H_2_O	CO_2_ (×10^–5^ cm^2^/s)	H_2_O (×10^–5^ cm^2^/s)	CN_CO2_ (1N)	CN_CO2_ (2N)	CN_CO2_ (3N)
Bulk	72	0	0.179 ± 0.003	/	0.77	0.61	0.06
HB-PEI^20^	72	72	0.042 ± 0.001	0.044 ± 0.002	0.71	0.51	0.06
Bulk	72	0	0.097 ± 0.002	/	0.63	0.45	0.04
HB-PPI^20^	72	72	0.017 ± 0.001	0.021 ± 0.002	0.60	0.33	0.02
Slab	72	0	0.311 ± 0.002	/	0.65	0.28	0.68
HB-PEI	72	72	0.125 ± 0.001	0.068 ± 0.002	0.57	0.29	0.67
Slab	72	0	0.181 ± 0.009	/	0.60	0.13	0.64
HB-PPI	72	72	0.074 ± 0.001	0.038 ± 0.001	0.64	0.74	0.59

Notably, the introduction of H_2_O leads to a universal decrease in CO_2_ diffusion across both environments, though transport rates remain comparatively higher in the slab. In bulk HB-PEI, the CO_2_ diffusion coefficient drops, upon hydration, from 0.179 to (0.042 ± 0.001) × 10^–5^ cm^2^/s upon hydration. A similar suppression is seen in bulk HB-PPI, where CO_2_ diffusion falls from 0.097 to (0.017 ± 0.001) × 10^–5^ cm^2^/s. Classical theory and experiments suggest that water should act as a plasticizer, enhancing polymer segmental chain mobility and facilitating CO_2_ diffusion by breaking up restrictive amine–amine networks; however, this behavior is typically characteristic of large-scale systems with longer polymer chains and higher free volume. In our smaller, constrained systems, this plasticization effect is insufficient to overcome the competitive dynamics at play; instead, water molecules act antagonistically by preferentially adsorbing to amine sites and forming extensive, competing hydrogen-bond networks that block CO_2_ accessibility. This trend is consistent with our previous study of neat, unsupported bulk HB-PEI membranes, where CO_2_ diffusivity was similarly found to decrease with hydration due to water occupying the free volume required for CO_2_ transport.^36^ We note that enhanced CO_2_ diffusion upon hydration, as observed in our related study of HB-PEI confined within silica MCM-41 pores,^19^ arises from a distinct, support-competition mechanism in which water preferentially displaces CO_2_ from strongly adsorbing silanol sites—a mechanism that is absent in the neat polymer systems studied here.

This competitive effect is even more pronounced at the slab interface, where reduced steric hindrance allows water to more readily access and occupy active sites. In slab HB-PEI, CO_2_ diffusion decreases from 0.311 to 0.125 ± 0.001 × 10^–5^ cm^2^/s upon hydration, while H_2_O itself diffuses more slowly still, at 0.068 ± 0.002 × 10^–5^ cm^2^/s, which indicates that even though water is more mobile than CO_2_ in the bulk, it becomes comparatively immobilized at the interface, consistent with strong, localized binding to surface amine groups. The same pattern is evident in slab HB-PPI, where CO_2_ diffusion falls from 0.181 to 0.074 ± 0.001 × 10^–5^ cm^2^/s, alongside an H_2_O diffusion coefficient of 0.038 ± 0.001 × 10^–5^ cm^2^/s.

This is directly supported by our pair correlation and coordination analyses: in bulk HB-PEI, the coordination numbers of CO_2_ with the amine sites decrease from 0.77/0.61/0.06 (dry) to 0.71/0.51/0.06 (hydrated) for the primary (1N), secondary (2N), and tertiary amines (3N), respectively, while in the slab, the corresponding values shift from 0.65/0.28/0.68 to 0.57/0.29/0.67 for the 1N, 2N, and 3N, respectively, demonstrating a more significant reduction in CO_2_–amine molecular association in the presence of water, particularly at the more accessible amine sites. In slab HB-PPI, by contrast, the 1N and 3N coordination numbers change only modestly upon hydration (0.60 → 0.64 and 0.64 → 0.59, respectively), whereas the 2N coordination number rises sharply from 0.13 to 0.74. This disproportionate increase suggests that, unlike in HB-PEI—where water directly competes with CO_2_ for secondary-amine sites—the secondary amines in HB-PPI, being less densely packed and more conformationally flexible at the interface, undergo a hydration-induced local reorganization that draws a larger population of CO_2_ into the secondary-amine solvation shell rather than displacing it. This behavior underscores a polymer-specific hydration response that becomes apparent only in the slab geometry.

Second, the slab models reveal a significant shift in binding-site preferences. In bulk simulations, CO_2_ predominantly coordinates with 1N and 2N, while 3N sites remain largely unutilized (coordination numbers < 0.1) when using the 4 Å cutoff. In contrast, the slab models demonstrate a dramatic increase in interactions with 3N, reaching coordination numbers as high as 0.68 in dry HB-PEI. As shown by the density profiles (Figure 3), tertiary amines preferentially segregate toward the polymer–air interface, whereas primary amines remain concentrated within the bulk-like core; it is this surface segregation—rather than the tertiary amines being more “interior”—that brings them into direct contact with the CO_2_-rich region near the surface. This indicates that the thin-film geometry alleviates the steric hindrance experienced by tertiary amines under fully periodic bulk conditions, allowing the highly branched, surface-segregated tertiary amines to interact more readily with CO_2_ molecules, thereby maximizing the overall CO_2_ capture efficiency of the hyperbranched networks.

This slab-induced enhancement of interfacial transport and amine accessibility is also supported by direct experimental measurements of PEI thin films. Using a tandem quartz crystal microbalance and polarization-modulation infrared reflection–absorption spectroscopy, Hoffman et al. studied planar PEI films of varying thickness and found that films thinner than 50 nm exhibited higher amine efficiency than thicker films, consistent with reduced diffusional resistance and greater accessibility of amine sites near the free surface.^50^ In a related study, the same group further showed that water vapor enhances CO_2_ capture specifically when uptake is limited by diffusional resistance, in agreement with our observation that the polymer–air interface promotes CO_2_ transport and amine utilization relative to the bulk.^51^

## 4 Conclusion

This study provides a quantitative and mechanistic understanding of CO_2_ capture in hyperbranched poly­(alkylene imine) thin films by explicitly incorporating polymer–air interfacial effects through slab-based molecular dynamics simulations. The results demonstrate that interfacial structure significantly alters both thermodynamic and transport properties compared to bulk systems. In particular, the introduction of a free surface increases the fractional free volume (FFV) from 19.1% to 25.0% in HB-PEI and from 19.9% to 29.9% in HB-PPI, accompanied by an increase in specific surface area from 0.27 to 0.29 Å^–1^ (HB-PEI) and from 0.29 to 0.34 Å^–1^ (HB-PPI). Despite these structural advantages in HB-PPI, transport analysis reveals that HB-PEI consistently exhibits higher CO_2_ diffusivity, with diffusion coefficients decreasing from 0.937 × 10^–5^ to 0.049 × 10^–5^ cm^2^/s under increasing CO_2_ and H_2_O loadings, whereas HB-PPI shows lower values ranging from 0.321 × 10^–5^ to 0.014 × 10^–5^ cm^2^/s.

This apparent discrepancy highlights that CO_2_ transport is governed not solely by geometric free volume but by polymer–gas interactions and segmental dynamics. Coordination number analysis further confirms that HB-PEI exhibits stronger CO_2_ affinity, with primary amine coordination decreasing from 0.655 to 0.464 under hydration, indicating competitive adsorption with water, while HB-PPI shows an increase from 0.600 to 0.716, reflecting distinct interaction mechanisms. Additionally, pair correlation analysis reveals preferential binding of CO_2_ to secondary amines in HB-PEI and primary amines in HB-PPI, emphasizing the role of local chemical environments. Importantly, hydration introduces a critical limitation by forming hydrogen-bonded networks and occupying free volume, leading to a substantial decrease in CO_2_ diffusivity (up to ∼95% in HB-PEI).

It is important to note that solid amine-based capture materials like HB-PEI and HB-PPI are well known to react through carbamate and bicarbonate mechanisms during the actual direct air capture (DAC) process. However, accurately describing these chemical reactions requires reactive molecular dynamics or ab initio molecular dynamics simulations, which are beyond the scope of this study. In addition, the present models were constructed using a single experimentally reported amine ratio; systematically varying the primary/secondary/tertiary amine composition to assess its impact on interfacial structure and CO_2_ transport represents an important direction for future work. With that in mind, these findings collectively establish that interfacial heterogeneity, amine distribution, and hydration effects synergistically determine the CO_2_ capture performance. Therefore, accurate prediction and optimization of DAC materials require explicit inclusion of interfacial models, as bulk-only approaches fail to capture these critical surface-mediated phenomena.

## Supplementary Material



## Notes

van der Waals interaction parameters for CO_2_–H_2_O and CO_2_–amine pairs (**Tables S1** and **S2**); Pair correlation functions for amine–H_2_O pairs in HB-PEI and HB-PPI slab systems under various configurations (**Figure S1**). Details of molecular model construction and equilibration of the slab models for hyperbranched poly­(ethylenimine) and hyperbranched poly­(propylenimine) systems (PDF)
