# Supplementary material for: CO2 Capture by Hyperbranched Poly(alkylene imine) Thin Films: A Molecular Dynamics Simulation Approach
Source: J Phys Chem B. 2026 Aug 26;130(36):9223–35. doi: 10.1021/acs.jpcb.6c02846 (PMC13573555; doi:10.1021/acs.jpcb.6c02846)
Supplement: Supplementary file 1 [file jp6c02846_si_001.pdf]

Supporting Information

CO_2 Capture of Hyperbranched Poly(Alkylene Imine) Thin Films: Molecular Dynamics Simulation Approach

Guilherme R. Weber Nakamura,[1,†] Junhe Chen,[1,†] Sung Hyun Kwon,[2,*] and Seung Soon Jang,[1*]

[1] Computational NanoBio Technology Laboratory, School of Materials Science and Engineering, Georgia Institute of Technology, 771 Ferst Drive NW, Atlanta, GA 30332-0245, USA

[2] School of Chemical Engineering, Pusan National University, 2 Busandaehak-ro 63beon-gil, Geumjeong-gu, Pusan, 46241, South Korea

[†] The co-first authors

[*] Corresponding authors:

Sung Hyun Kwon sunghyun.kwon@pusan.ac.kr

Seung Soon Jang seungsoon.jang@mse.gatech.edu

orcid.org/0000-0002-1920-421X

Keywords: CO_2 Capture, Hyperbranched Poly(ethylene imine), Hyperbranched Poly(propylene imine), Force Field, Molecular Dynamics Simulation

Table S1. van der Waals Interaction Parameters for CO_2-H_2O pair

**Force Field Type**		r_0	D
O_F3C	C_1	3.03021	0.59295
H_F3C	C_1	2.75949	0.98243
C_1	C_1	3.87252	0.09264
C_1	O_2	3.01418	0.10666
O_2	O_2	3.42393	0.09575
O_F3C	C_3N	3.55263	0.57769
O_F3C	N_3	3.20737	0.17560
O_F3C	H_	2.81499	0.07534
O_F3C	H___A	3.34354	0.00642
H_F3C	C_3N	2.15083	0.01715
H_F3C	N_3	2.05270	0.05437
H_F3C	H_	1.70819	0.03035
H_F3C	H___A	2.20941	0.00108

Table S2. van der Waals Interaction Parameters for CO_2-Amine pair

**Force Field Type**		r_0	D
C_1	C_3N	3.33189	0.09669
C_1	N_3	3.36700	0.08000
C_1	H_	3.24220	0.04507
C_1	H___A	3.00154	0.00403
O_2	C_3N	3.41241	0.11184
O_2	N_3	3.09141	0.77512
O_2	H_	3.10920	0.03960

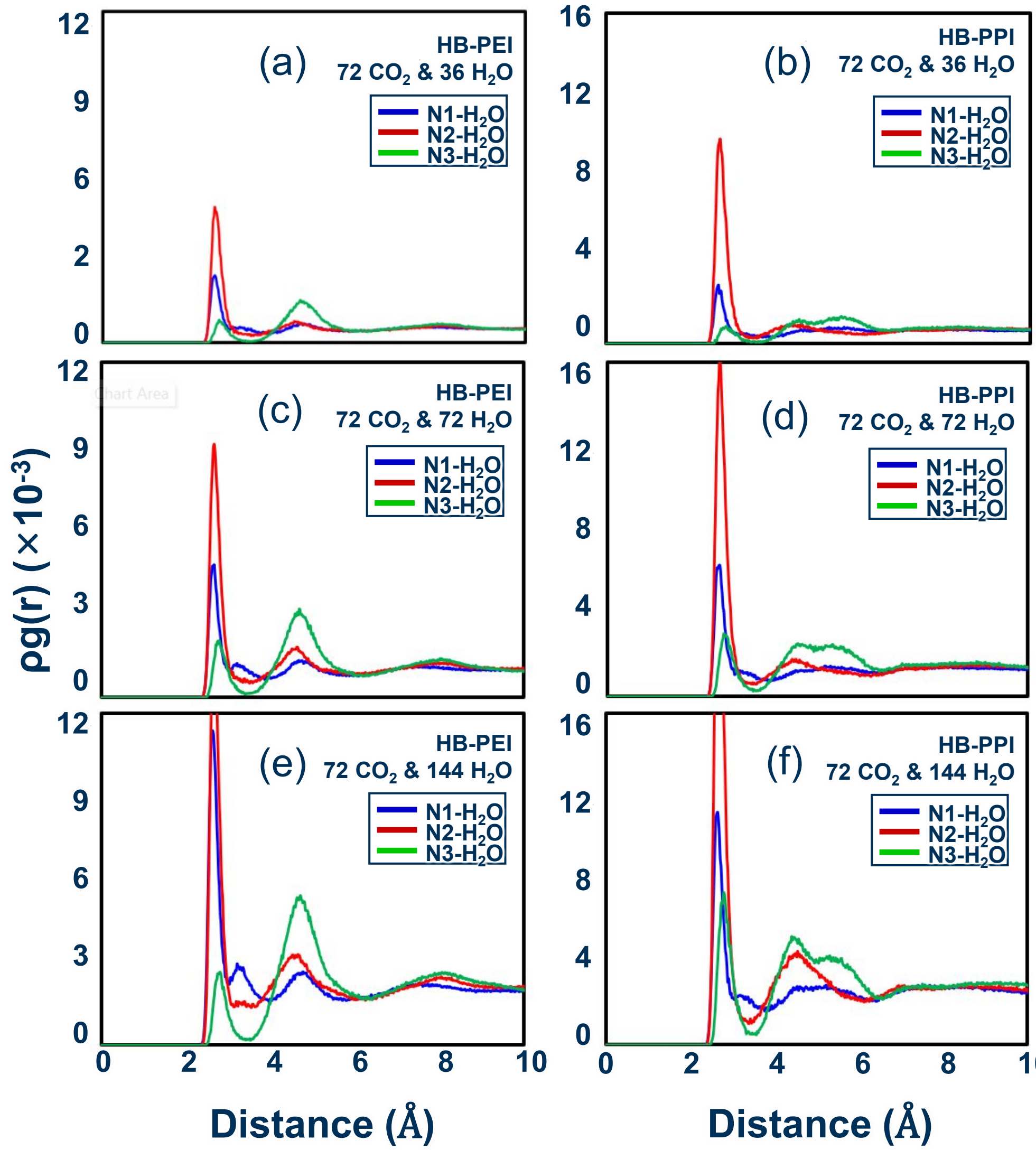

Figure S1. Pair correlation functions for amine-H_2O pairs in HB-PEI and HB-PPI slab systems: (a, b) for PEI-72-36 and PPI-72-36, respectively; (c, d) for PEI-72-72 and PPI-72-72, respectively; (e, f) for PEI-72-144 and PPI-72-144, respectively.

Molecular Model Construction and Equilibration

Atomistic models of slab hyperbranched poly(ethylenimine) (HB-PEI) and slab hyperbranched poly(propylenimine) (HB-PPI) were constructed following the systematic framework. In this method, polymer backbones were explicitly assembled using a stepwise monomer connection algorithm that reflects the known branching architecture of each polymer. For HB-PEI, aziridine-based repeating units were linked to form a hyperbranched structure with varying generations of primary, secondary, and tertiary amines. Similarly, HB-PPI structures were generated using iterative Michael addition and reduction steps mimicking commercial dendritic architectures. Each final structure contained a fixed number of nitrogen atoms and amine types in a ratio consistent with the theoretical generation.

The initial polymer chains were randomly inserted into a 40 × 40 × 40 $Å^3$ periodic simulation box, ensuring no atom overlaps. The systems were then equilibrated through a multistage procedure: (1) energy minimization using a steepest descent algorithm to eliminate high-energy contacts, (2) high-temperature NVT annealing at 600 K for 1 ns to relax the polymer conformations, and (3) subsequent NPT equilibration at 1 atm and 300 K for 5 ns. Convergence at this stage was assessed by confirming energy and density plateaued visually within the final 1 ns of the NPT run. Following this initial equilibration, the simulation box was used as the starting configuration for the thermal annealing procedure described in Section 2.2 (following Jang and Goddard), which was applied prior to the production 60 ns NVT simulation. Then, the 40 × 40 × 40 $Å^3$ simulation box was inserted into the center of a 40 × 40 × 120 $Å^3$ simulation box, periodic in the x- and y-axes only, with empty space above and below the slab of HB-PEI and HB-PPI, and this relaxation procedure was repeated. All simulations were performed using the LAMMPS package with a time step of 1 fs and an Nosé–Hoover Chain thermostat.

Total, kinetic, and potential energy curves, along with other thermodynamic variables, were monitored to verify convergence. This protocol ensures that the amorphous configurations used in subsequent CO_2 and H_2O simulations reflect physically reasonable and thermodynamically stable structures.
